# Unique resistance traits against downy mildew from the center of origin of grapevine (*Vitis vinifera*)

**DOI:** 10.1038/s41598-018-30413-w

**Published:** 2018-08-21

**Authors:** Silvia Laura Toffolatti, Gabriella De Lorenzis, Alex Costa, Giuliana Maddalena, Alessandro Passera, Maria Cristina Bonza, Massimo Pindo, Erika Stefani, Alessandro Cestaro, Paola Casati, Osvaldo Failla, Piero Attilio Bianco, David Maghradze, Fabio Quaglino

**Affiliations:** 1 Università degli Studi di Milano, Dipartimento di Scienze Agrarie e Ambientali - Produzione, Territorio e Agroenergia (DiSAA), via Celoria 2, 20133 Milano, Italy; 2 Università degli Studi di Milano, Dipartimento di Bioscienze (DBS), via Celoria 26, 20133 Milano, Italy; 3 Fondazione E. Mach, Centro Ricerca e Innovazione, Via E. Mach 1, 38010 San Michele all’Adige, (TN) Italy; 4 Scientific - Research Center of Agriculture, Marshal Gelovani Avenue 6, 0159 Tbilisi, Georgia; 5 Faculty of Agricultural Sciences and Biosystems Engineering, Georgian Technical University, David Guramishvili Avenue 17, 0175 Tbilisi, Georgia

## Abstract

The Eurasian grapevine (*Vitis vinifera*), an Old World species now cultivated worldwide for high-quality wine production, is extremely susceptible to the agent of downy mildew, *Plasmopara viticola*. The cultivation of resistant *V. vinifera* varieties would be a sustainable way to reduce the damage caused by the pathogen and the impact of disease management, which involves the economic, health and environmental costs of frequent fungicide application. We report the finding of unique downy mildew resistance traits in a winemaking cultivar from the domestication center of *V. vinifera*, and characterize the expression of a range of genes associated with the resistance mechanism. Based on comparative experimental inoculations, confocal microscopy and transcriptomics analyses, our study shows that *V. vinifera* cv. Mgaloblishvili, native to Georgia (South Caucasus), exhibits unique resistance traits against *P. viticola*. Its defense response, leading to a limitation of *P. viticola* growth and sporulation, is determined by the overexpression of genes related to pathogen recognition, the ethylene signaling pathway, synthesis of antimicrobial compounds and enzymes, and the development of structural barriers. The unique resistant traits found in Mgaloblishvili highlight the presence of a rare defense system in *V. vinifera* against *P. viticola* which promises fresh opportunities for grapevine genetic improvement.

## Introduction

The Eurasian grapevine (*Vitis vinifera*) is one of the most extensively cultivated crops with worldwide economic importance, being the only grapevine species used for the production of quality wine. *V. vinifera* is characterized by high genetic and phenotypic variability, but selection carried out by humans has limited cultivation to a reduced number of varieties selected for their yielding capacity and traits related to quality, such as berry composition, phenology and resistance to stresses, capturing only part of the diversity of the species^1^. The *V. vinifera* varieties that are prevalently cultivated globally are susceptible to various pathogens responsible for severe crop losses, including the Oomycete *Plasmopara viticola*, the causal agent of downy mildew. This pathogen has a polycyclic behaviour and infects all the green parts of the host leading to quantitative yield losses, due to direct infection of the berries, as well as qualitative damage^2^. The threat posed by this fungal pathogen, combined with the ineffectiveness of agronomic practices in halting its diffusion, makes frequent spraying of fungicides unavoidable in areas experiencing high disease pressure. This contributes to the status of viticulture as the agricultural activity which makes the most intensive use of plant protection products (http://ec.europa.eu/eurostat/en/web/products-statistical-books/-/KS-76-06-669). This leads not only to negative impacts on farmers’ economic situations, human health and the environment, but also to a potential reduction of future disease control due to the development of fungicide resistance^3^. For these reasons, the cultivation of pathogen-resistant grapevine varieties is one of the most straightforward strategies to reduce the impact of plant protection against *P. viticola* while maintaining the quantity and quality of yield. Nonetheless, this process is complicated by genetic factors: *Vitis* accessions that co-evolved with the pathogen and show partial or total resistance to *P. viticola*, originating from North America (*e.g. V. labrusca, V. aestivalis, V. riparia*) and Asia (*e.g. V. amurensis*), can be used as sources of resistance genes in breeding programs^2^. However, the use of these non-*vinifera* parents, which are less suitable for winemaking, often introduces unwanted traits in the offspring during breeding, resulting in a serious reduction of wine quality. This important limitation of breeding programs could be avoided by employing resistance genes of *V. vinifera* origin, but until recently no indigenous varieties have been shown to exhibit these traits. In fact, previous studies suggested that *V. vinifera* does not have a specific response to infection compared to the American grapevines^4,5^.

The South Caucasian region is characterized by a rich biodiversity of *V. vinifera* cultivars and by the presence of numerous wild grapevine populations, the ancestral forms of cultivated species^6^. In the South Caucasus, Georgia is considered to be the original center for winemaking and grapevine domestication^7^. Recent studies showed that sources of resistance in *V. vinifera* are indeed present at the phenotypic level in Georgian wild and cultivated germplasm^6,8,9^.

In this study, we focus on Mgaloblishvili, the *V. vinifera* autochthonous cultivar from Georgia^10^ that showed the most promising phenotype in terms of resistance against *P. viticola* in an initial screening study^9^. Comparative experimental inoculations, confocal microscopy and transcriptomics analyses allowed the description of a rare defense system against *P. viticola* in winemaking *V. vinifera*, distinct from that of American *Vitis* species, opening new perspectives for sustainable viticulture through improved breeding programs.

## Results and Discussion

The behavior of Mgaloblishvili in response to *P. viticola* was primarily compared to that of the susceptible variety Pinot noir, for characterizing the resistance mechanism in *V. vinifera*. Finally, Mgaloblishvili was compared to the resistant interspecific hybrid variety Bianca for pointing out the differences between the resistance mechanisms of *V. vinifera* and American grapevine.

### Mgaloblishvili reduces disease severity by deregulating *P. viticola* growth and sporulation

To characterize the interaction of *P. viticola* with the *V. vinifera* cultivar Mgaloblishvili, pathogen structures, disease severity and sporangia production were evaluated at 1–3 and six days after inoculation (dai) of leaf tissues with the pathogen and compared with the same parameters measured for susceptible cultivar Pinot noir.

**Figure 1:**
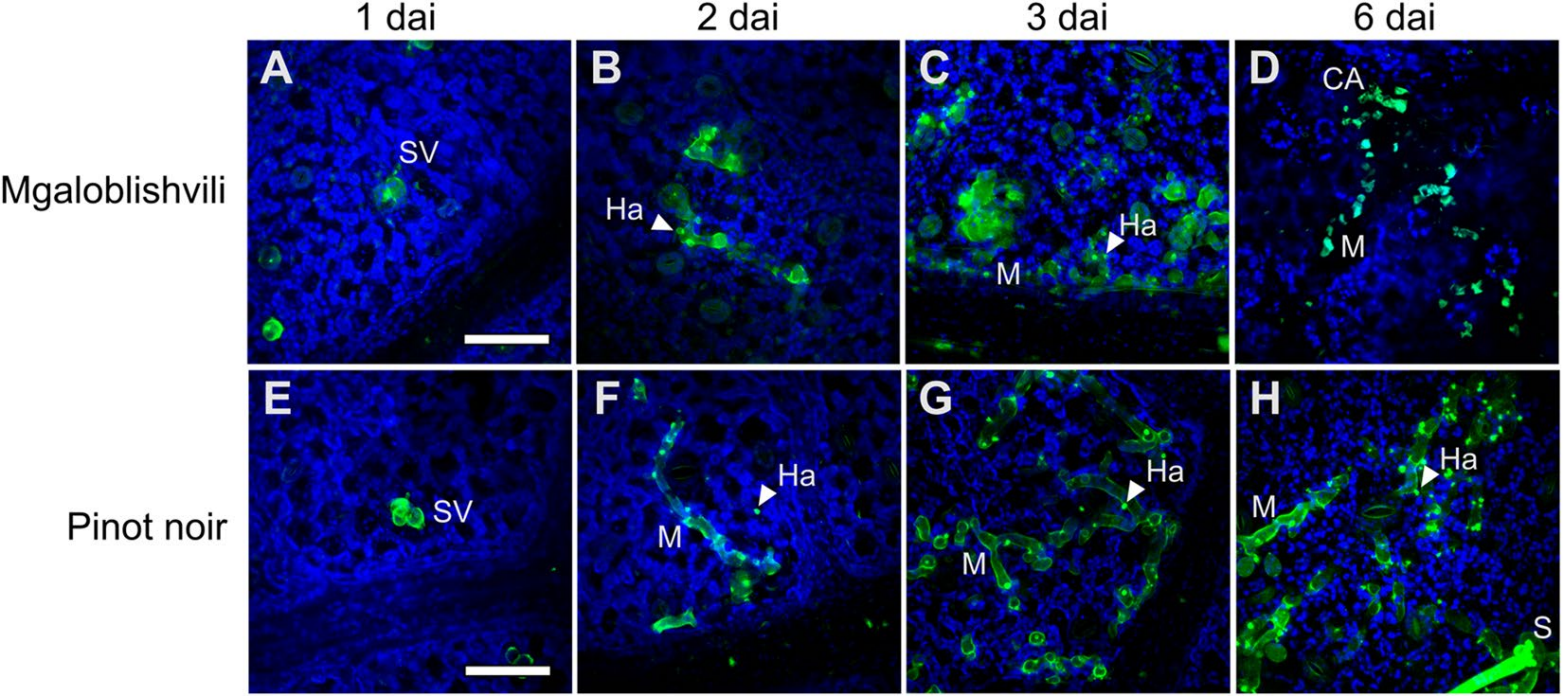
Time course colonization of Mgaloblishvili (**A**–**D**) and Pinot noir (**E**–**H**) leaves by *P. viticola* at 1 (**A**,**E**), 2 (**B**,**F**), 3 (**C**,**G**) and 6 (**D**,**H**) days after inoculation (dai) visualized through confocal microscopy. *SV = substomatal vesicle; M = mycelium; Ha = haustorium; S = sporangiophore; CA = callose deposition. Green: aniline blue staining; blue: chlorophyll. Scale bar: 50 μm.

**Figure 2:**
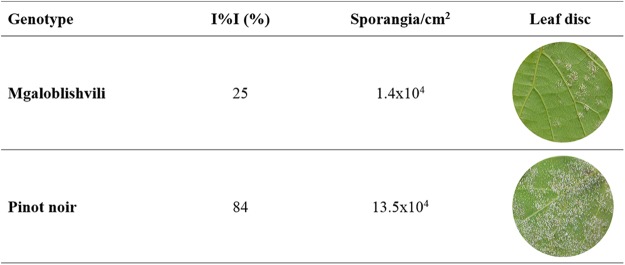
Average values of disease severity (I%I) and sporangia density (Sporangia/cm^2^) recorded on the leaf discs of Mgaloblishvili and Pinot noir inoculated with *P. viticola* at 6 dai (days after inoculation). Pathogen sporangiophores and sporangia are visible in white on the lower surface of leaf discs.

Three dimensional (3D) confocal microscopy analyses were carried out following aniline blue staining to establish the growth of pathogen within leaf tissues of Mgaloblishvili and Pinot noir. The analyses showed that the colonization of leaf tissues by *P. viticola* followed an analogous pattern in the two cultivars until two dai: the asexual spores of the pathogen penetrated through the stomatal pore and differentiated the substomatal vesicle (Fig. 1A,E) from which the primary hypha with haustorium originated (Fig. 1B,F). Starting from 3 dai, notable differences in pathogen development were observed between Mgaloblishvili and Pinot noir. While *P. viticola* growth inside the leaf tissues of Pinot noir (Fig. 1G,H) showed the regular pattern described by other authors^5^, evident alterations were visible in Mgaloblishvili: *P. viticola* hyphae were hyper-branched, contorted (Fig. 1C) and ill defined, indicating a loss of integrity of the vegetative structure. At 6 dai, dead portions of mycelium, surrounded by callose barriers (Fig. 1D), and short, hyperbranched and partly sterile sporangiophores emerging from the stomata were visible in Mgaloblishvili (Fig. S1). At the same time point, disease severity on inoculated leaf discs of Mgaloblishvili was significantly reduced (ANOVA; F = 62.6; df = 1–4; *P* = 0.001), being 3.4 times lower than on Pinot noir. Analogously, sporulation per leaf unit showed a significant nine-fold reduction (F = 9.7; df = 1–4; *P* = 0.03), indicating that the plant employs defense reactions to reduce pathogen infection and sporulation in comparison with Pinot noir (Fig. 2). These results confirm suggestions from previous studies obtained on Mgaloblishvili plants cultivated mainly under field conditions, hence underlining its resistance to *P. viticola*^9^. The overall reduction in the ability of the pathogen to colonize leaf tissues and reduced sporangium production indicate that the plant defense reaction consisted of the synthesis of physical barriers, involving callose encapsulation, leading to the degeneration of large portions of mycelium and the alteration of sporangiophore shape. Callose deposition is typical of the resistance reactions of several grapevine species resistant to *P. viticola*, such as *Muscadinia rotundifolia*, *V. pseudoreticulata* and *V. amurensis*^11,12^. Similar alterations in the sporangiophores were observed by other authors following different treatments such as exposure to continuous white light^13^ or chemical treatment with 2-deoxy-D-glucose, which interferes with glucan synthesis^14^. The deregulation of *P. viticola* sporulation can have particularly important consequences under field conditions, because the lesser occurrence of inoculum can contribute towards slowing the progress of disease epidemics^15^.

### Transcriptome analysis overview

Whole transcriptome analysis was performed on inoculated and non-inoculated leaves of Mgaloblishvili, Pinot noir and Bianca collected at three time points (1, 2 and 3 dai). The trimmed reads were mapped on the PN40024 12X v2 grape reference transcriptome. The average number of unique mapping reads per sample was ~52 million, ranging from 48 to 55 million reads, and a percentage of 78% successfully mapped reads (from 73 to 82% of reads). The similar percentages of mapped reads overall the *V. vinifera* and non-*vinifera* samples confirmed the suitability of PN40024 12X v2 grape reference transcriptome for read mapping of non-*vinifera* genotypes, such as the *Vitis* interspecific crossing Bianca and other interspecific hybrids^16–19^.

The overview of count table has been represented by heatmap and hierarchical analysis (Fig. S2). The samples were clustered in three main groups: (i) some of the inoculated and non-inoculated Mgaloblishvili and Pinot noir samples collected at 0 and 1 dai; (ii) the majority of Bianca inoculated and non-inoculated samples; (iii) the majority of Mgaloblishvili and Pinot noir inoculated and non-inoculated samples. The highest correlation was obtained among samples of the same variety, as well as among replicates collected from inoculated and non-inoculated plants. The PCA (Principal Component Analysis) plot showed a good correlation among biological replicates (Fig. S3). The first two components accounted for 71% of total variance and identified three main groups based on the variety. Overall, PC1 (Principal Component 1) differentiated Bianca from the other two varieties, while the PC2 differentiated Mgaloblishvili and Pinot noir samples. Bianca showed the most homogeneous replicates, for both inoculated and non-inoculated conditions. Both hierarchical analysis and PCA showed a good correlation according to variety.

Differentially expressed genes (DEGs) obtained by comparing inoculated and non-inoculated samples of each cultivar are listed in the Table S1. No statistically significant differences were observed among inoculated and non-inoculated samples at 0 dai in all genotypes. The total number of DEGs (sum of DEGs at 1, 2, and 3 dai) recorded in the cultivars was different: Bianca showed the highest number (6393 DEGs), followed by Pinot noir (2748 DEGs), and the least amount (1432 DEGs) was detected in Mgaloblishvili (Table S2). Likewise, a difference of total DEGs at the different time points (sum of DEGs for Mgaloblishvili, Pinot noir and Bianca at 1, 2, or 3 dai) can be noticed: the highest number of DEGs was recorded at 1 dai, followed by 3 dai, and the least amount was scored at 2 dai; in particular, Mgaloblishvili showed no DEGs at 2 dai (Table S2). This evidences that the three varieties reprogrammed their cellular mechanisms early upon inoculation with *P. viticola*.

At 1 dai, the three genotypes shared a core of 181 DEGs, and the highest number of DEGs (774) was shared between Bianca and Pinot noir, followed by Mgaloblishvili and Pinot noir (600 DEGs), and Bianca and Mgaloblishvili (550 DEGs). At 2 dai, Bianca and Pinot noir shared two DEGs, while at 3 dai the three cultivars shared seven DEGs (Fig. S4A).

Moreover, at each time point, cultivar-specific DEGs were found: for example, in Mgaloblishvili 28% (371 out of 1340, at 1 dai) and 64% (89 out of 139, at 3 dai) of total DEGs were not shared with Pinot noir nor Bianca (Fig. S4A). Finally, four and 31 DEGs were constantly identified in the considered time points in Pinot noir and Bianca, respectively (Fig. S4B).

### Gene expression patterns of Mgaloblishvili under infection

To investigate the resistance response mechanism in *V. vinifera*, the gene expression patterns of Mgaloblishvili and Pinot noir induced by *P. viticola* inoculation were analyzed and compared. The highest number of DEGs was detected in the two varieties following inoculation with *P. viticola* at 1 dai. Subsequently, the number of DEGs strongly decreased, reaching the lowest value at 2 dai and increasing a little at 3 dai (Table 1, Fig. S4). This trend, already described in previous studies^4,5^, is thought to be correlated with the infection process. The plant usually responds to *P. viticola* infection after contact between the first haustorium and the plant cell membrane^20,21^, which occurs within 1 dai. This was also observed in the present study (Fig. 1A,E). Consequently, we showed the results of the subsequent analyses carried out on the DEGs observed at 1 dai, unless otherwise specified.

**Table 1:** Overview of differentially expressed genes (nr. and %) detected in Mgaloblishvili, Pinot noir and Bianca at three different time points after leaf inoculation with *P. viticola*.

Cultivar	Differentially expressed genes					
	T1		T2		T3	
	Nr.	%	Nr.	%	Nr.	%
**Mgaloblishvili**						
*Up*	575	0.79	—	—	83	0.02
*Down*	765	0.56	—	—	56	0.05
**Pinot noir**						
*Up*	1015	3.70	13	0.07	256	0.90
*Down*	1363	4.90	10	0.04	218	0.82
**Bianca**						
*Up*	3635	11.00	107	0.47	454	0.47
*Down*	2266	7.40	30	0.15	436	0.15

T1 = 1 day after inoculation (dai), T2 = 2 dai, T3 = 3 dai.

**Figure 3:**
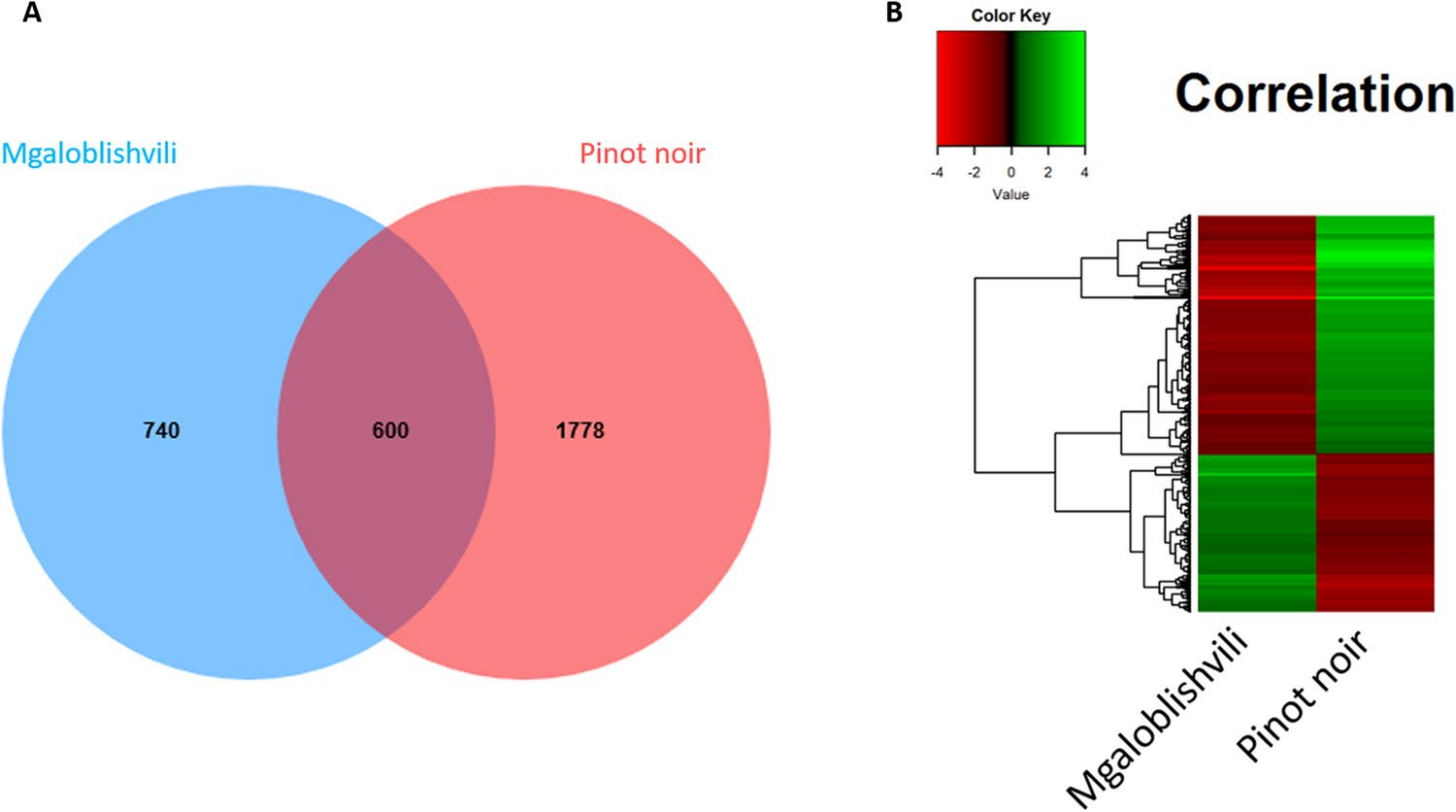
Comparison between differentially expressed genes (DEGs) of Mgaloblishvili and Pinot noir at 1 day after inoculation with *P. viticola*. (**A**) Venn diagram; (**B**) Heatmap of 600 DEGs shared by Mgaloblishvili and Pinot noir. Green: upregulated genes; red: downregulated genes.

To identify how the Mgaloblishvili transcriptome was affected by *P. viticola* infection, we mainly focused on the 600 DEGs shared with Pinot noir and the 740 DEGs unique to Mgaloblishvili (Fig. 3A). With regard to DEGs shared between Pinot noir and Mgaloblishvili, the heatmap (Fig. 3B) highlighted that upregulated genes in one cultivar were downregulated in the other and *vice-versa*. In Pinot noir, pathogen inoculation induced a downregulation of genes involved in the plant defense mechanism from 1 dai, which was not detected in a previous study^5^.

A GO enrichment assay, including the top 50 GO terms (Table S2), was performed to identify the biological processes mostly affected by *P. viticola* infection. The most highly upregulated genes in Mgaloblishvili are involved in plant responses to stimuli and stress factors, signal transduction, protein ubiquitination, and metabolism of glycoproteins. To elucidate the Mgaloblishvili response to *P. viticola*, DEGs were filtered for log_2_ FC values above 1.5, yielding 38 genes differentially expressed only in Mgaloblishvili and 58 DEGs shared with Pinot noir (Table S3A,B). The putative role of these genes in response to *P. viticola* will be described in the following paragraphs.

### Mgaloblishvili recognizes *P. viticola* through specific receptors

Oomycetes undergo a series of developmental stages throughout a successful infection cycle, including the formation of sporangia, release of motile zoospores, their encystment and germination to form hyphae, haustoria and, finally, sporangiophores^2^. The development of pathogen inside the host leads it to be constantly in contact with the host plasma membrane which includes pattern recognition receptors (PRRs) that identify pathogen-associated molecular patterns (PAMPs), molecules which are essential for microbes to establish an infection^22^. *P. viticola* PAMPs include β-glucans and cell wall components, and recognition occurs *via* the invading haustorium^23^. Laminarins in particular have been shown to elicit a defense reaction in *V. vinifera*^24^.

Several genes encoding for receptor kinases known to be involved in recognizing PAMPs at the extracellular interface and leading to pathogen resistance^25^ were strongly overexpressed in Mgaloblishvili upon *P. viticola* inoculation (Table S3). Among these are: two G-type lectin receptor kinases and a cysteine-rich RLKs (CRK1), three leucine-rich repeat protein kinases, and two serine/threonine protein kinases. Interestingly, genes encoding for receptor kinase proteins with a role in cell wall integrity sensing (DAMP: damage-associated molecular patterns) and in pathogen perception and resistance, such as Wall-Associated Kinase (WAK) and MDIS1-interacting receptor like kinase 2 (MIK2)^26,27^, were also over-expressed. Of the above cited genes, four were downregulated in Pinot noir. This suggests that Mgaloblishvili primarily exploits receptors of extracellular signals to establish the resistance response.

Successful pathogens use effector molecules, encoded by *Avr* genes, not only to suppress host immunity but also to manipulate host cellular mechanisms for their own benefit, leading to effector-triggered susceptibility (ETS)^28^. An increasing number of effectors has been characterized for Oomycetes^28^. Based on homology sequences, the presence of a wide array of effectors, supposedly interfering with the response of the *V. vinifera* defense mechanism, has also been detected in the *P. viticola* genome, with extensive differences between European and Chinese strains^29^. To counteract the action of the pathogen, plants have evolved specific receptors called NBS-LRR (nucleotide-binding site leucine-rich repeat), proteins encoded by resistance *R* genes, able to recognize the effectors and ultimately activating defense mechanisms leading to effector-triggered immunity (ETI) through hypersensitive response (HR)^30^. Among the known NBS-LRRs is the rust resistance Lr10 locus, encoding for CC-NBS-LRR receptors involved in wheat leaf rust resistance^31^. Surprisingly, in the present study several isoforms of the Lr10 gene were found to be overexpressed in Mgaloblishvili, indicating that this cultivar could be capable of specifically recognizing fungal effectors. In contrast, a strong downregulation of these gene isoforms was found in Pinot noir, highlighting the uniqueness of Mgaloblishvili in this respect. Since Mgaloblishvili exploits genes encoding for PAMPs receptors and effectors upon *P. viticola* inoculation, it seems likely that the plant activates PAMP-triggered immunity (PTI) and an incomplete ETI response. In fact, the wide array of effectors present in the *P. viticola* genome, along with the upregulation of isoforms of a single NBS-LRR receptor, could explain why the resistant phenotype is not related to HR in Mgaloblishvili.

Recently, it has been demonstrated that ubiquitination in plant cells modulates signaling mediated by PAMP receptors and is important for the accumulation of NBS-LRR receptors^32^. Several RING H2-type E3 ligases, involved in ubiquitination process, are activated in response to biotic and abiotic stresses. The upregulation of genes encoding for RING-H2 ATL proteins is related to *P. viticola* resistance in non-*vinifera* grapevine species. In contrast, these genes are downregulated in susceptible *V. vinifera* cultivars^33^. This is confirmed by the results obtained in the present study for Pinot noir, where two genes encoding for RING-H2 ATL proteins (ATL39 and ATL20) were downregulated. For the first time in a *V. vinifera* cultivar, Mgaloblishvili has been demonstrated to exhibit a gene encoding for a RING-H2 finger protein-like, namely ATL22, which was strongly upregulated on *P. viticola* inoculation (Table S3A). The expression of this given gene has not been affected in Pinot noir, suggesting its putative involvement in Mgaloblishvili resistance.

### The defense mechanism of Mgaloblishvili is mediated by ethylene

The plant defense mechanism is regulated by a complex network of signaling transduction pathways^34,35^. In particular, jasmonic and salicylic acid signaling, modulated by gibberellins^34^, are associated with the defense response against the downy mildew agent in resistant non-*vinifera* grapevines^5,36^. In Mgaloblishvili, the overexpression of gibberellin 2-beta dioxygenase 2 gene (Table S3A), involved in gibberellin catabolism, indicates that this system is probably not involved in the resistance response. In contrast, the overexpression of several genes associated with ethylene signaling, such as genes encoding for ethylene-responsive transcription factor elements 5 and 1B (Table S3B) and GDSL esterase/lipase like 1 (Table S3A), indicates that the defense mechanism can be mediated mainly by ethylene. Interestingly, GDSL LIPASE-LIKE 1 (GLIP1) plays an important role in plant immunity, eliciting both local and systemic resistance in plants^37^. Indeed, other ethylene-regulated genes, encoding for NAC (Table S3A,B), WRKY and β-glucanase (Table S3B), were differentially induced by *P. viticola* infection, providing confirmation. Among NAC genes, one NAC transcription factor and two NAC-domain containing-proteins were identified, which are known to be induced by the exogenous application of ethylene, wounding and pathogen infections. The response of NAC genes to *P. viticola* infection was in accordance with other studies, indicating their potential as early regulators in the response to ethylene and other hormone signaling^38^. Similarly, the WRKY transcription factors have been shown to bind the promoter elements of PR genes and to modulate their expression^39^. β-glucanases are thought to be PR proteins and were shown to be ethylene-responsive and to exhibit antifungal activity both *in vitro* and *in planta*. They function both with an indirect antifungal effect, involving fragmentation of fungal cell wall (rich in chitin and glucans) to release elicitors that induce plant defense responses, and by a direct effect derived from digestion of glucan fibers and the resulting weakening of fungal cell walls^40^. The modulation of β-glucanases has previously been detected in response to artificial inoculation with *P. viticola* in *V. riparia* and *V. vinifera*^27^. Interestingly, the analysis of promoter regions for NAC, WRKY and β-glucanase genes revealed the presence of stress-responsive *cis*-elements, such as GCC-box (data not shown), known as recognition sites for ethylene-responsive transcription factors^41^.

Genes related to the IAA signaling system were also upregulated, but later than the ethylene pathway. Indeed, tryptophan aminotransferase-related protein gene (Table S3A,B), leading to IAA synthesis, was upregulated at 1 dai but genes encoding for IAA-induced proteins have been identified only at 3 dai (Table S1).

### Mgaloblishvili overexpresses genes for antimicrobial compounds and structural barriers

Among pathways regulated by ethylene-signaling, the phenylpropanoid pathway leads to the synthesis of compounds with antimicrobial and antioxidative activity, such as terpenoids, flavonoids and stilbenes^5,42,43^. Interestingly, genes implicated in the synthesis of terpenoids, *i.e*. several cytochrome P450s (Table S3) and a geraniol 8-hydroxylase (Table S3B)^44,45^, and flavonoids, *i.e*. an aureusidin synthase (Table S3B)^46^, were extensively overexpressed in Mgaloblishvili. Terpenes are known to exhibit activity against phytopathogenic fungi, herbivore attack and abiotic stress^47,48^. Aurones belong to a class of flavonoids synthesized during plant-pathogen interaction^5,42,43^.

In *Vitis* spp., stilbenes are accumulated in response to various biotic and abiotic stresses, including pathogen attack^49^. In Mgaloblishvili, the expression of genes involved in the stilbene biosynthesis is not differentially modulated by *P. viticola* infection.

Structural defenses, such as cell wall reinforcement and callose deposits, are an important part of the plant defense mechanism. The cell wall is a barrier that hinders pathogen invasion and its alteration acts as a source of signaling to activate the plant defense response^50^. Consequently, the strengthening of cell wall occurs through a secondary cell wall deposition characterized by cross-linked racemic lignin macromolecules and small amounts of pectins and xyloglucans^51^. In Mgaloblishvili, *P. viticola* does not penetrate the cell wall until haustorium formation, which occurred by 1 dai (Fig. 1A). The penetration of plant cell wall by the pathogen caused the overexpression of genes associated with cell wall integrity perception (MIK2 upregulation; Table S3A) and the transition from primary to secondary wall synthesis through the upregulation of a cellulose synthase-like protein G3 gene (Table S3C)^52^ and the downregulation of xyloglucan endotransglycosylase genes (Table S1)^53^. None of these changes occurred in Pinot noir (Tables S1 and S3).

### Validation of candidate genes expression by real-time RT-PCR

Differential expression of five candidate genes representative of each part of the putative resistance mechanism (recognition, signaling and resistance response) was validated through Real-time RT-PCR (Table S4) on Mgaloblishvili and Pinot noir samples. A highly significant correlation was found between the RNA-seq and real-time RT-PCR results (*P* = 0.001, R^2^ = 0.9114). PCA explained almost all variability among samples (90%) and clearly distinguished the two varieties on the principal component 1 (PC1** = **66%). PC2 (24%) partially identifies a shift between inoculated and non-inoculated samples, but this difference was also partly explained by PC1 (Fig. S5). These results suggest that the investigated genes are indeed related to the response of the two cultivars to *P. viticola*.

### Mgaloblishvili exhibits unique features compared to a resistant hybrid

Finally, Mgaloblishvili response to *P. viticola* was compared to that of a reference resistant variety, Bianca, to explore the possible existence of shared or disparate mechanisms underpinning resistance. Bianca is an interspecific hybrid selected and cultivated in Hungary for wine production and obtained through a long and complex hybridization process. The parentage of Bianca is approximately 80% *V. vinifera* and 20% of non-*vinifera*, with a genetic background originating from other resistant *Vitis* species, such as the American *V. labrusca*, *V. rupestris*, *V. berlandieri* and *V. lincecumii*^54^. Resistance to *P. viticola* in grapevine Bianca is controlled by a major dominant gene, associated with localized HR in leaf tissues immediately after pathogen infection, preventing mycelial growth and sporulation within host tissues^55^. Resistance based on single resistance genes (qualitative resistance) generally induces a strong selection pressure on pathogen populations and, as a consequence, a rapid increase in the frequency of strains that are able to overcome the host defence reactions. Indeed, resistance breaking isolates of *P. viticola* have been found in Bianca^21,55^.

Differences between the two cultivars in response to pathogen were observed in experimental inoculations and confocal microscopy analyses: while deregulation of *P. viticola* growth was found in Mgaloblishvili (Fig. 1A–D), HR occurred in Bianca (Fig. S6). RNA-seq analyses revealed the presence of 550 DEGs shared by the two cultivars and 790 DEGs unique to Mgaloblishvili (Fig. S4). To compare the response of Mgaloblishvili and Bianca to *P. viticola*, DEGs were filtered for log_2_ FC values above 1.5, yielding 7 DEGs upregulated in both cultivars and 38 DEGs upregulated only in Mgaloblishvili (Table S3C,D).

The common upregulated DEGs are involved in signal transduction (calcium-binding protein), synthesis of antimicrobial compounds (cytochrome P450 78A4 element) and structural defenses (cellulose synthase-like protein G3)^56^.

Remarkable differences in the defense mechanism, associated with unique characteristics of Mgaloblishvili, were found in genes related to pathogen recognition, signaling and antimicrobial compound synthesis (Table S3D). The most relevant genes encoded for: numerous receptor protein kinases, including putative receptor-like kinases Lr10; a β-glucosidase, putatively acting as elicitors for the production of signaling molecules^57^, and a BTB/POZ domain-containing protein involved in the indirect activation of disease resistance protein PR1, associated with Systemic Acquired Resistance^58^; enzymes involved in the synthesis of antimicrobial compounds, such as a cytochrome P450 (CYP72A219 element), and a valencene synthase, involved in the biosynthesis of valencene^59^, a sesquiterpene acting in the inhibition of hyphal growth and zoospore viability in Oomycetes^60^.

## Conclusions

**Figure 4:**
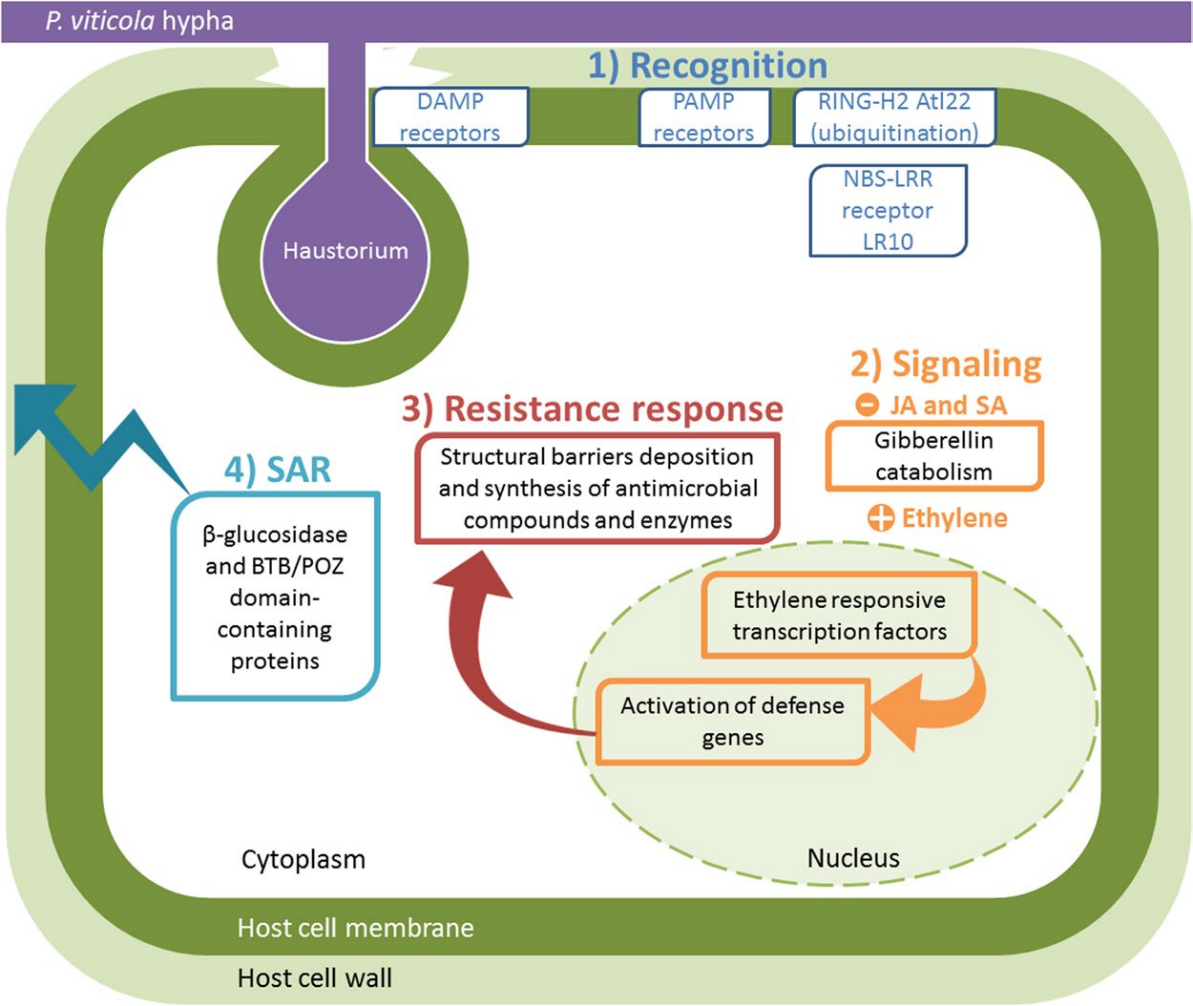
Schematic representation of the putative resistance mechanism of Mgaloblishvili (in green) against *P. viticola* (in purple) based on the overexpression of genes involved in the plant defense pathway at 1 dai. (**1**) Pathogen recognition through PAMP, DAMP and effector receptors, and ubiquitination. (**2**) Phytohormone signaling based on ethylene. (**3**) Resistance response based on synthesis of antimicrobial compounds and fungal wall degradating enzymes, and cell wall reinforcement. (**4**) Systemic signaling based on the indirect activation of the disease resistance protein PR1 that is involved in Systemic Acquired Resistance (SAR).

The results reported in this work show for the first time the existence of resistance pathways against one of the most important grapevine biotic stresses, downy mildew, within the *V. vinifera* germplasm of Georgia, previously explored for its resistance or tolerance to other important pathogens, such as phytoplasmas^61,62^. The putative defense response observed in Mgaloblishvili, schematically represented in Fig. 4, is determined by the overexpression of genes downregulated in a susceptible *V. vinifera* cultivar and exhibiting unique features compared to those of an American grapevine, which relies on HR. The response to *P. viticola* is associated not only with PAMP and DAMP recognition but also with a weak effector recognition, mediated by Lr10 locus, not leading to HR. This could be compatible with the absence of co-evolution between *P. viticola* and Mgaloblishvili, resulting in an inability to recognize all the effectors of the pathogen. *P. viticola* was in fact introduced into Europe from Northern America in 1878, reaching South Caucasus in the last decade of the century^6,63^. The historic time period since pathogen introduction has apparently been too brief to allow co-evolution between the pathogen and plant, an effect that is undoubtedly exacerbated by the asexual vegetative propagation of this crop plant. Overall, the defense mechanism is mainly mediated by the ethylene signaling pathway and consists of the limitation of *P. viticola* growth and sporulation through the expression of genes involved in the synthesis of antimicrobial compounds, mainly terpenoids and flavonoids but also glucanases affecting pathogen cell wall integrity, and in the deposition of structural barriers that both thicken the host cell wall and encapsulate hyphae in a callose-like material.

The identification of resistance traits within the *V. vinifera* gene pool is an unique precedent, opening new and promising perspectives for sustainable disease management of grapevine because it can lead to: (i) a more durable resistance through the exploitation of new target genes for *V. vinifera* genetic engineering (ii) changes in the strategies adopted for disease resistance breeding, which can now concentrate on the Eurasian grapevine as an easier method than hybridization with American grapevines; (iii) screening of other *V. vinifera* varieties to detect novel resistant genotypes; (iv) increased efforts for biodiversity conservation, to preserve Georgian and other local grapevine genetic resources as a sources of useful traits to be exploited in additional branches of viticulture.

Generally speaking, the detection of resistance to downy mildew in our study, and to powdery mildew^63^ and phytoplasmas^61^ in previous studies, demonstrates that the Eurasian grapevine *V. vinifera* is more capable of responding to biotic stresses than was previously known or believed. This confirms yet again the importance of *V. vinifera* gene pool from the more ancient area of grapevine cultivation, extending from South Caucasus to Central Asia.

## Methods

### Plant material and experimental inoculation with *P. viticola*

Two-year-old plants of the Georgian *V. vinifera* variety Mgaloblishvili, the international *V. vinifera* variety Pinot noir, and the *Vitis* interspecific hybrid variety Bianca were grown in greenhouse (24 °C, 16 h photoperiod, 70% relative humidity) at the Department of Agricultural and Environmental Sciences (Milan, Italy) in 5 L pots filled with sand-peat mixture (7:3 v/v), regularly watered *via* a drip system and fertilized twice a year with Osmocote Topdress fertilizer (ICL Specialty Fertilizers, Italy).

Four plants per variety were used for the experiment. For each plant, two shoots were used in the experimental procedure: the first one was spray-inoculated with the pathogen and the second one was sprayed with sterile distilled water. Experimental inoculations were carried out by spraying a sporangia suspension of *P. viticola* prepared as described by Toffolatti and coworkers^9^, on the underside of the 2^nd^–5^th^ leaves starting from the apex of the shoots. 2.5 × 10^4^ *P. viticola* sporangia were sprayed per leaf. Shoots were covered with transparent plastic bags to keep humidity high and to favor the infection process where needed.

### Sample collection and preparation

Three leaves (biological replicates) per variety (Mgaloblishvili, Pinot noir and Bianca) and treatment (inoculated/non-inoculated with *P. viticola*) were randomly collected at 0, 1, 2 and 3 days after inoculation (dai). A leaf disc (2 cm diameter) was cut from each sample for confocal microscopy analysis, and the rest of the leaf tissues was frozen at −80 °C for RNA extraction. The leaf discs were fixed in 0.1 M phosphate buffer (pH 7.2) containing 2% paraformaldehyde and 3% glutaraldehyde and kept at 4 °C. All microscopy reagents were purchased from Sigma-Aldrich (Italy).

Furthermore, three inoculated leaves were sampled at 6 dai for assessing disease severity (I%I), evaluating the number of sporangia formed by the pathogen^21^, and performing microscopy observations. ANOVA was performed on I%I and sporangia/cm^2^ to evaluate the existence of significant differences among cultivars (SPSS v. 24, IBM Analytics Italia, Italy).

### Confocal microscopy

The pathogen development was observed on samples prepared from leaves collected at 1, 2, 3, and 6 dai on Mgaloblishvili, Pinot noir and Bianca. Leaf discs were stained with 0.05% aniline blue in 0.067 M K_2_HPO_4_ (pH 9) for 24 hours^21^. This dye was selected as its chemical properties allow it to bind with β-1,3-glucans present both in the cell wall of the pathogen and in the callose deposition produced by plant cells in response to infection^64,65^. Confocal microscopy analyses were performed using an inverted microscope, Leica DMIRE2 with a HCX APO L U-V-I 63.0 × 0.90 water immersion objective, equipped with a Leica TCS SP2 laser scanning device (Leica, Germany). To detect aniline blue fluorescence, leaves were excited by the 405 nm UV laser and the emission was collected between 450/530 nm. For chlorophyll detection, samples were excited at 514 nm line of the Argon laser and the emission was collected between 650/750 nm. Images were captured as z-series (50–60 stacks spaced every 0.5 µm) of optical sections (3-D), keeping the same parameter settings in all acquisitions, and displayed as single maximum-intensity projections (MIPs) obtained by Fiji, an open-source platform for biological-image analysis^66^. MIPs were obtained for the fluorescence of both targets and then merged to obtain the images shown in Figs 1, S1 and S6.

### RNA extraction

RNA was extracted from 100 mg of sampled tissues. Samples wer ground with liquid nitrogen and RNA was extracted using the Spectrum™ Plant Total RNA Kit (Sigma-Aldrich), according to the manufacturer’s instructions.

RNA was quantified using Qubit® RNA HS Assay Kit by Qubit® 3.0 Fluorometer (Life Technologies, CA); its integrity and purity (260/230 and 260/280 ratios) were assessed using respectively Agilent RNA 6000 Nano Kit on an Agilent 2100 Bioanalyzer (Agilent Technologies, CA), and NanoDrop Spectrophotometer (Thermo Scientific, MA). The samples showing a 260/230 ratio lower than 1.8 were subjected to lithium-chloride purification^67^.

### Library construction and sequencing

Starting from 1 μg of high quality total RNA per sample, 72 cDNA libraries were constructed according to the KAPA Stranded mRNA-Seq Kit (Kapa Biosystems, MA). Each library was barcoded using the SeqCap Adapter kit A and B (Roche NimbleGen, WI) and the final size of 250–280 bps was confirmed on High Sensitivity D1000 ScreenTape by Tapestation 2200 (Agilent). All the libraries were quantified by KAPA Library Quantification kit – Illumina (Kapa Biosystems) using the LightCycler 480 (Roche, Switzerland), and randomly multiplexed in seven pools in equimolar way. Each pooled library was sequenced on an Illumina HiSeq2500 platform (Illumina, CA) with paired end runs of 2 × 50 bps. Base calling and quality control were performed on the Illumina RTA v1.13 (Illumina) sequence analysis pipeline. The original sequencing datasets have been deposited in the European Nucleotide Archive (ENA) with the accession number PRJEB24540.

### Sequence annotation

Raw reads quality was visually inspected by means of FastQC software^68^ and then processed to remove low quality bases and contaminants (Illumina adapters). Cleaning phase was then performed with Trimmomatic software version 0.36^69^ using an average quality cut-off of 30 (Phred score) and a minimum read length of 40 bp. Cleaned reads were then mapped against predicted mRNAs (PN40024 12X v2 grape reference transcriptome) obtained from the gene prediction version 2.0 of the National Centre for Biotechnology Information. Mapping was performed using Bowtie2^70^ tool with default parameters. Alignments were first converted in a binary alignment map (BAM), a binary representation of the Sequence Alignment/MAP (SAM), and then sorted and indexed for the count of reads per mRNA, using SAMtools^71^ software package. The number of reads aligning to each transcript were counted using an *ad hoc* Python script.

### Statistical analysis of differentially expressed genes (DEGs)

To have an overview of similarities and dissimilarities among samples, the count data were used to perform heatmap analysis with hierarchical clustering and Principal Component Analysis (PCA) with DESeq2 R package^72^. Transcripts with less than 5 reads were not included in the analysis. To determine the differentially expressed genes (DEGs) between the different treatments (inoculated *vs* non-inoculated samples) of each variety at each time point, a multifactor designs method has been performed with DESeq2 R package. For each transcript, the log_2_ fold change (FC), *p*-value and adjusted *p*-value were evaluated, and only RefSeq IDs with false discovery rate (FDR)-adjusted *p*-value < 0.05 were retained. The protein sequence and functional information of DEGs were obtained from UniProt database^73^ or InterProScan tool for functional domain identification^74^.

Two and three-way Venn diagrams showing the overlaps among different time points per genotype were calculated and built using the jVenn web-server^75^, using DEGs as input data. The graphical representation of heatmap, hierarchical clustering and Principal Component Analysis (PCA) were performed using the function *heatmap.2* implemented in *gplots* R package^76^.

### Gene ontology (GO) enrichment analyses

Gene ontology (GO) enrichment analyses were performed with R package topGO version 2.26.0^77^. Enrichment test was run on DEGs showing a FDR-adjusted *p*-value < 0.05 and gene2GO annotation file was retrieved by the Grape genome browser of CRIBI Center^78^. A classical enrichment analysis was performed by testing the over-representation of GO terms within the group of DEGs, using the statistical Fisher’s exact test. The top-50 significantly enriched GO IDs were listed and recorded based on the terms of biological process ontology.

### Real-time reverse transcriptase-PCR

Expression of five upregulated genes representative of the resistance mechanism of Mgaloblshivili to *P. viticola* at 1 dai, was investigated through semi-quantitative real-time reverse transcriptase (RT)-PCR analyses (Table S4).

The cDNA of inoculated and non-inoculated Mgaloblishvili and Pinot noir samples was synthesized starting from 800 ng of total RNA using random nonamers and 200 U of M-MLV reverse transcriptase (Thermo Scientific), according to the manufacturer’s instructions. The cDNA was diluted 1:1 with sterile water and amplification reactions were carried out in three technical replicates per sample.

Gene specific primers (Table S4) were designed using Primer3 Plus software^79^. Ubiquitin^80^ and actin^81^ genes were used as references for data normalization. Real-time RT-PCRs were performed using a SYBR green method on a StepOne Plus Real-Time PCR System (Thermo Scientific) thermal cycler. Each 10 μl PCR reaction contained 0.6 μl of each primer (10 μM), 1 μl of diluted cDNA, 1X SYBR Green Real-Time PCR Master Mix (Thermo Scientific) and sterile water. Thermal cycling conditions were: 95 °C for 10 min followed by 40 cycles of 94 °C for 15 s, 58 °C for 30 s, and 72 °C for 30 s, and a melt cycle with 1 °C increments from 55 to 96 °C. The expression of each gene in different varieties and treatments was calculated by comparing their 2^−ΔΔCt^ values^82^. Such values were employed in a PCA analysis (SPSS) revealing the collective differential expression patterns of the five genes. Linear correlation between the differential expression levels of the five genes obtained by RNA-seq and real-time RT-PCR analyses was tested in SPSS. Goodness of fit was evaluated through significance and R^2^ values.

## Electronic supplementary material

Supplementary information

Table S1

Table S2

Table S3
