# Supplementary material for: Unique resistance traits against downy mildew from the center of origin of grapevine (Vitis vinifera)
Source: Sci Rep. 2018 Aug 21;8:12523. doi: 10.1038/s41598-018-30413-w (PMC6104083; doi:10.1038/s41598-018-30413-w)
Supplement: Supplementary file 1 — Supplementary information [file 41598_2018_30413_MOESM1_ESM.doc]

**Unique resistance traits against downy mildew from the center of origin of grapevine (*Vitis vinifera*)**

Silvia Laura Toffolatti^a^*, Gabriella De Lorenzis^a^*, Alex Costa^b^, Giuliana Maddalena^a^, Alessandro Passera^a^, Maria Cristina Bonza^b^, Massimo Pindo^c^, Erika Stefani^c^, Alessandro Cestaro^c^, Paola Casati^a^, Osvaldo Failla^a^, Piero Attilio Bianco^a^, David Maghradze^d,e^, Fabio Quaglino^a^

^a^Università degli Studi di Milano, Dipartimento di Scienze Agrarie e Ambientali - Produzione, Territorio e Agroenergia (DiSAA), via Celoria 2, 20133, Milano, Italy

^b^Università degli Studi di Milano, Dipartimento di Bioscienze (DBS), via Celoria 26, 20133, Milano, Italy

^c^Fondazione E. Mach, Centro Ricerca e Innovazione, Via E. Mach 1, 38010 San Michele all’Adige (TN), Italy

^d^Scientific - Research Center of Agriculture, Marshal Gelovani Avenue 6, 0159. Tbilisi. Georgia

^e^Faculty of Agricultural Sciences and Biosystems Engineering, Georgian Technical University, David Guramishvili Avenue 17, 0175, Tbilisi, Georgia.

*These authors equally contributed to the work; to whom correspondence should be addressed.

Email: [silvia.toffolatti@unimi.it](mailto:silvia.toffolatti@unimi.it); [gabriella.delorenzis@unimi.it](mailto:gabriella.delorenzis@unimi.it)

Supporting Information

**Figure S1.** *P. viticola* sporangiophores (S) and sporangia (Sp) emerging from the stomata of Mgaloblishvili leaves at 6 days after inoculation and visualized through confocal microscopy. A) Pathogen structures highlighted in green following aniline blue staining. B) Chlorophyll in blue. C) Merged pathogen structures and chlorophyll. *M= mycelium. Scale bar: 50 μm.


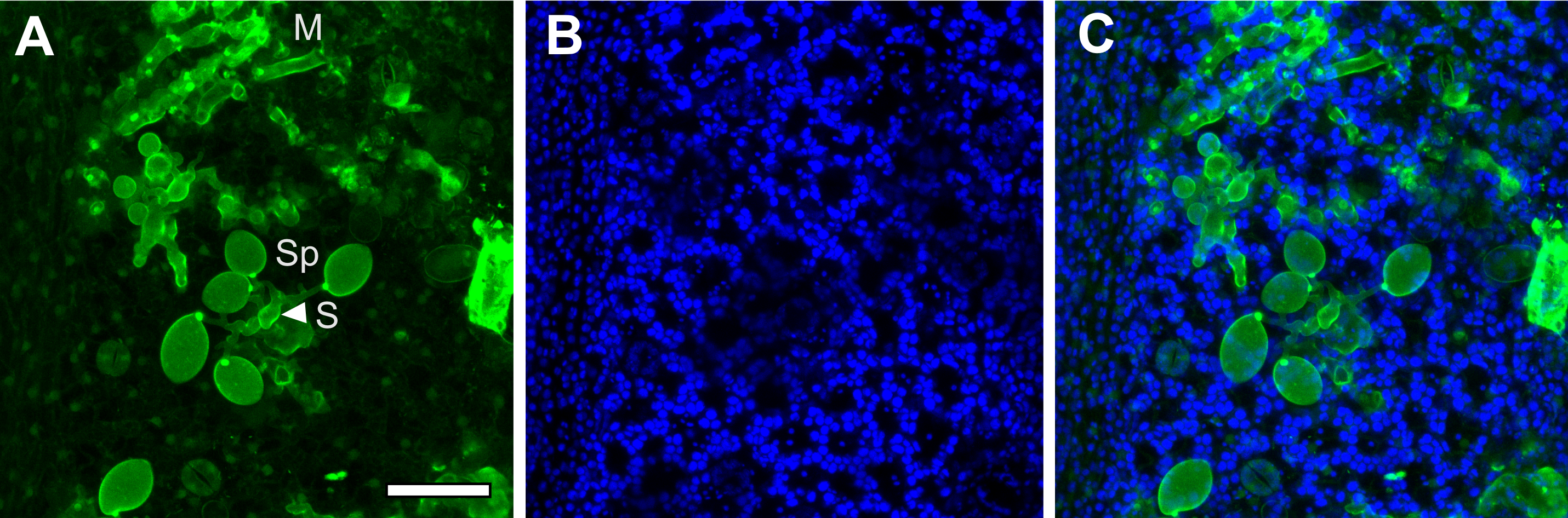


**Figure S2.** Overall representation of changes in mRNA-Seq reads of three grapevine genotypes (Mgaloblishvili, Pinot noir and Bianca) inoculated and non-inoculated with *P. viticola* at three different time points. T0 = 0 days after inoculation (dai), T1 = 1 dai, T2 = 2 dai, T3 = 3 dai. Heatmap and hierarchical clustering were obtained to explore the count table of mRNA-Seq reads.


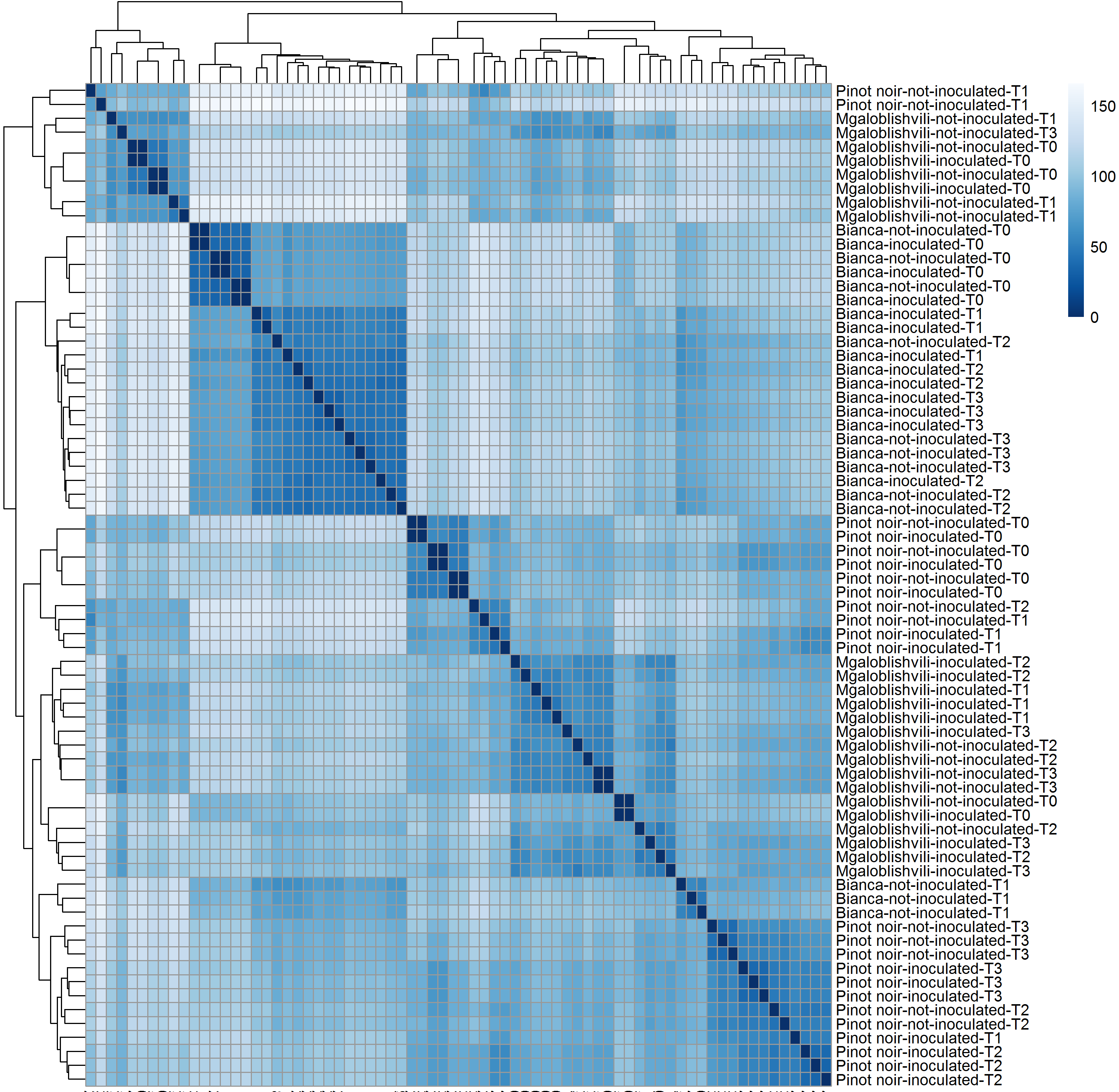


**Figure S3.** PCA (Principal Component Analysis) plot of the overall mRNA-Seq read count table of three grapevine genotypes (Mgaloblishvili, Pinot noir and Bianca) inoculated and non-inoculated with *P. viticola*. PC1: Principal Component 1; PC2: Principal Component 2.


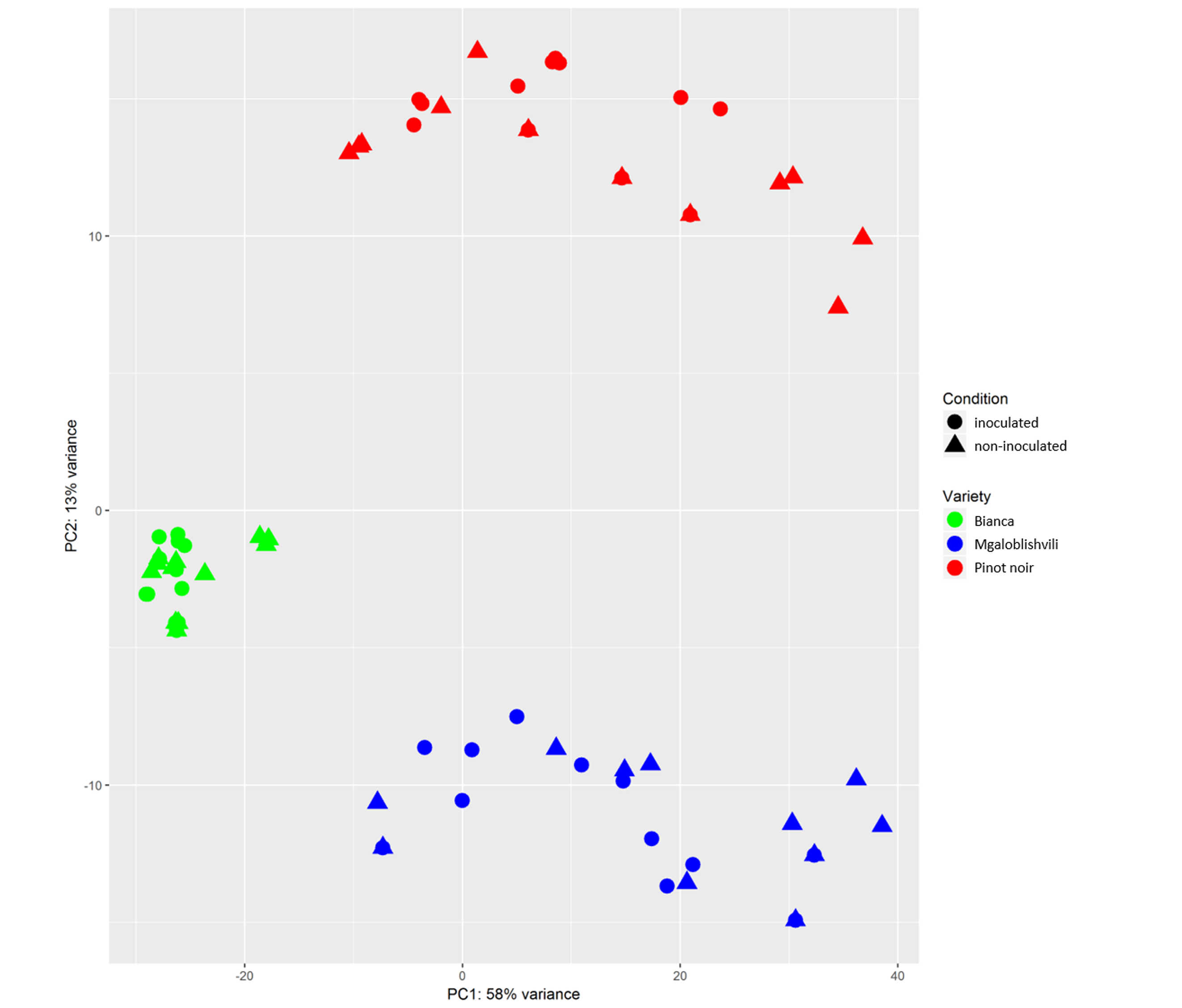


**Figure S4.** Venn diagrams illustrating differentially expressed genes (DEGs) among three time points after inoculation with *P. viticola*. A) Comparison among three grapevine cultivars at each time point. B) Comparison among DEGs at each time point per cultivar. PN = Pinot noir, M = Mgaloblishvili and B = Bianca. T1 = 1 day after inoculation (dai), T2 = 2 dai, T3 = 3 dai.


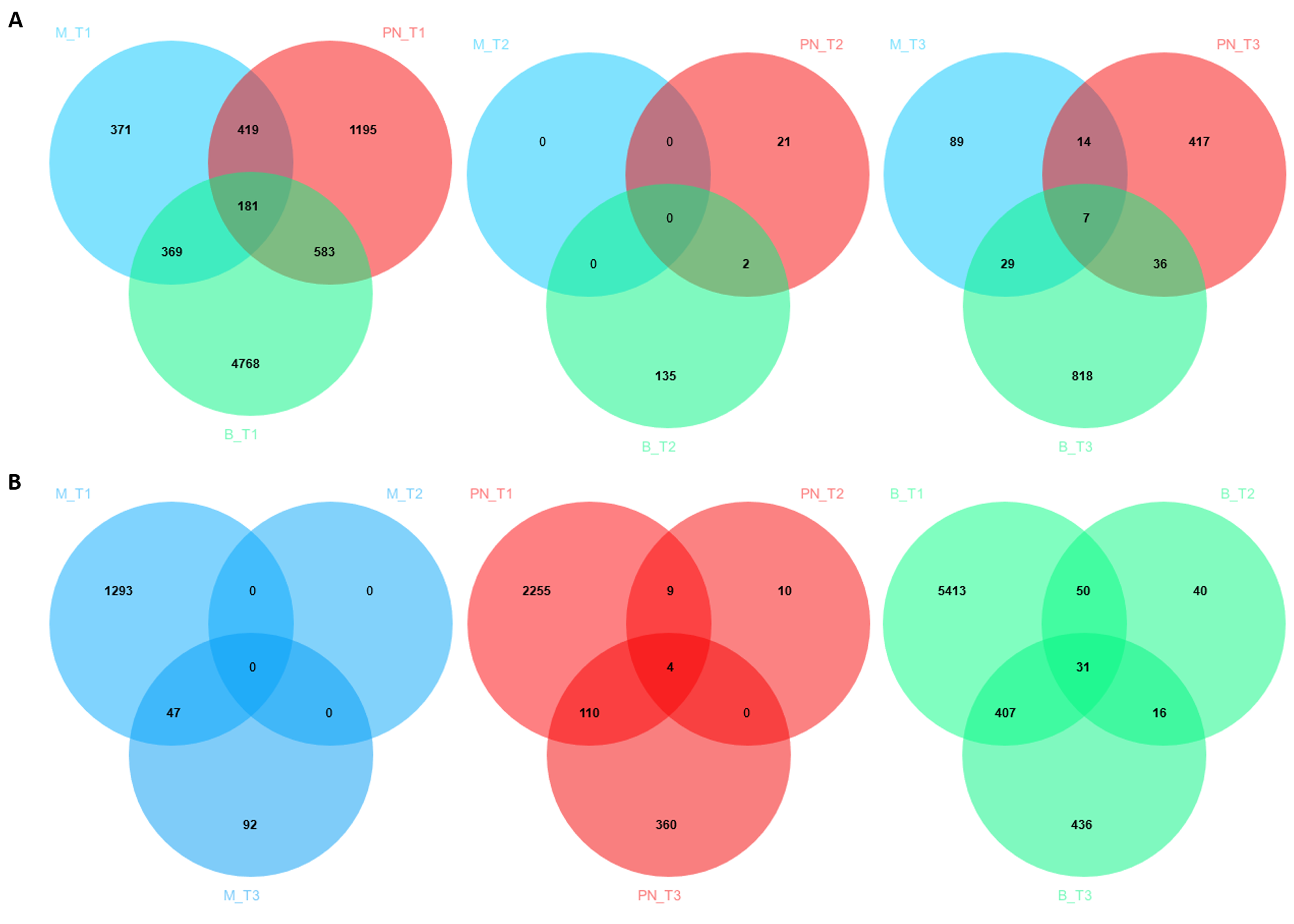


**Figure S5.** PCA plot based on real-time RT-PCR gene expression of 5 differentially expressed genes between two grapevine genotypes (Mgaloblishvili and Pinot noir) inoculated with *P. viticola* (at 1 day after inoculation) and non-inoculated. PC1: Principal Component 1; PC2: Principal Component 2. Percentage: variance.

**
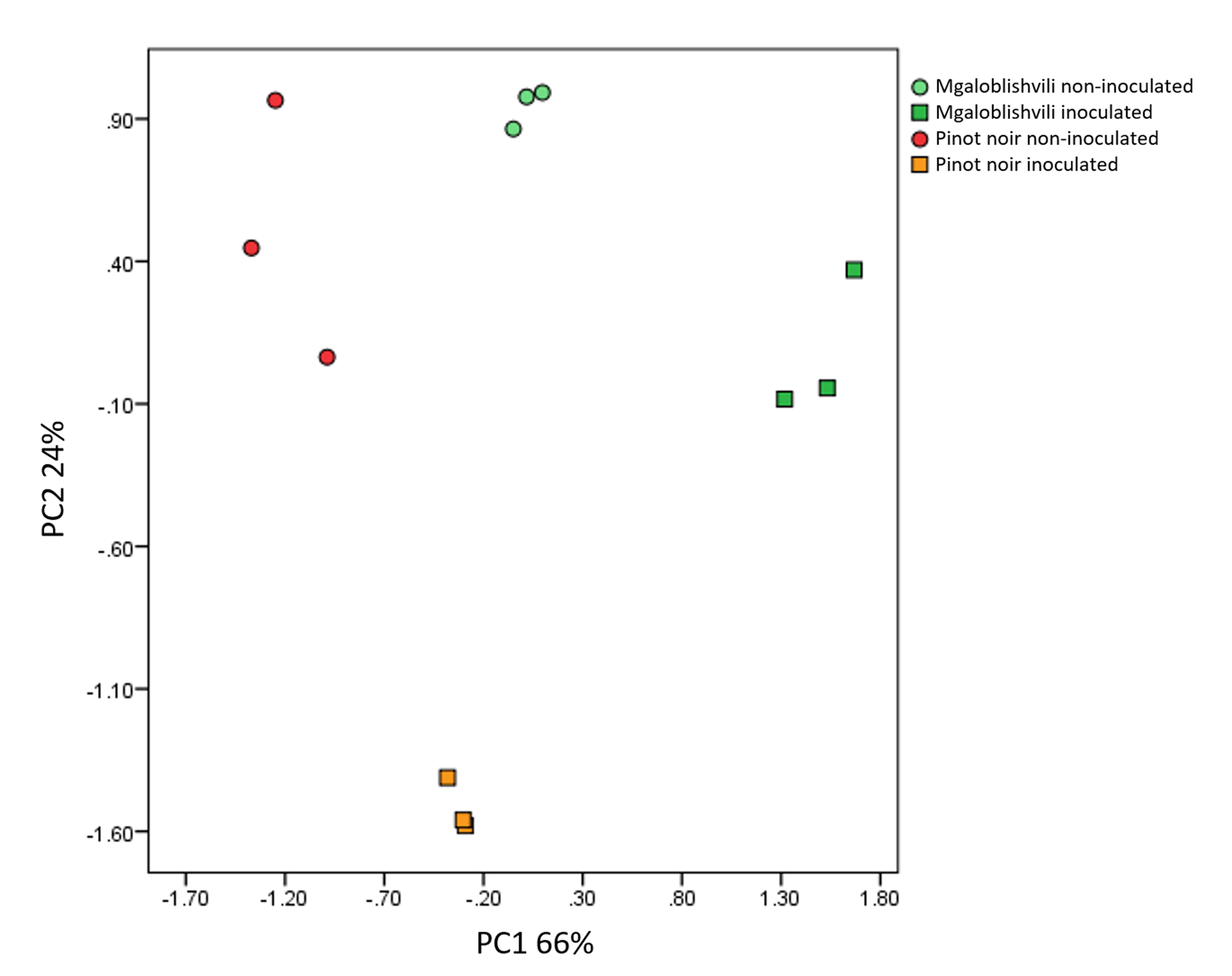
**

**Figure S6.** Time course colonization of Bianca leaves by *P. viticola* visualized through confocal microscopy at times: 1 (A, B, C) and 6 (D) days after inoculation (dai). Hypersensitive response, associated with callose deposition, occurred from 1 dai (B), blocking pathogen development (A, C) and was visible to the naked eye as localized necrosis (brown spots) on the leaf disc at 6 dai (E). *SV = substomatal vesicle; CA = callose deposition. Green: aniline blue staining; blue: chlorophyll. Scale bar: 50 μm.


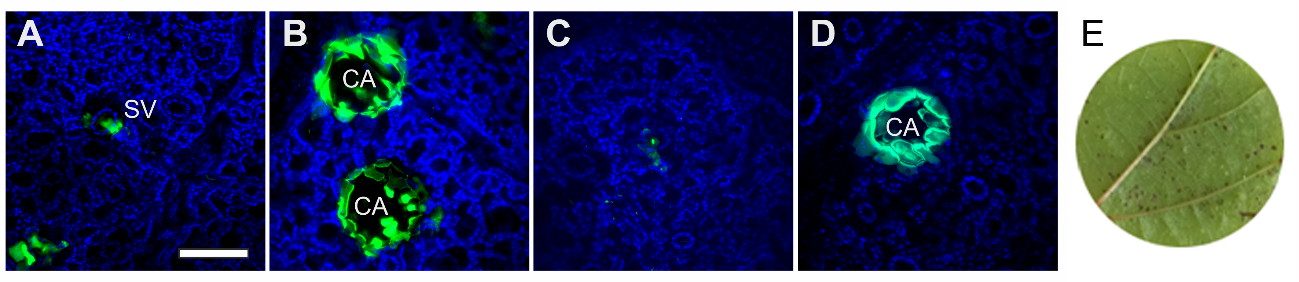


**Table S1.** List of differentially expressed genes identified in leaves of three grapevine cultivars (Mgaloblishvili, Pinot noir and Bianca) upon inoculation with *P. viticola*. T1 = 1 day after inoculation (dai), T2 = 2 dai, T3 = 3 dai.

(see Excel file)

**Table S2.** List of top 50 GO categories related to up- and downregulated differentially expressed genes for Mgaloblishvili, Pinot noir and Bianca at 1 day after *P. viticola* inoculation.

(see Excel file)

**Table S3.** List of differentially expressed genes (DEGs) upregulated with log_2_ fold change above 1.5 in Mgaloblishvili leaves at 1 day after *P. viticola* inoculation. Sheet A: DEGs upregulated in Mgaloblishvili alone. Sheet B: DEGs upregulated in Mgaloblishvili and downregulated in Pinot noir. Sheet C: DEGs upregulated in Mgaloblishvili and Bianca. Sheet D: DEGs upregulated in Mgaloblishvili and downregulated in Bianca.

(see Excel file)

**Table S4**. List of five pairwise primers used for quantitative real-time RT-PCR analysis.

| **Gene ID** | **Putative protein names** | **Primer name** | **Primer sequence (5’ 🡪 3’)** | **Amplicon size (bp)** |
| --- | --- | --- | --- | --- |
| XM_002271918.2 | cytochrome P450 87A3 | CYT87-F | TGGCAAACTCCCTCCAGG | 196 |
|  |  | CYT87-R | AGTAGTTGAAGTCTGGGTCCG |  |
| XM_002284498.2 | MLP-likeprotein 34 | M34-F | TGATTGGAAGAGTGTGGGCT | 192 |
|  |  | M34-R | CCGTCATCCTTTGCACTCAC |  |
| XM_002275661.3 | endo-1,3;1,4-beta-D-glucanase | EGLU-F | ATTCTACGTGGTGGTCCCTG | 168 |
|  |  | EGLU-R | AATCCTGCTGCTCCAATTGC |  |
| XM_002263215.3-  XM_002267534.3 | Rust resistance kinase Lr10 | RK10-F | AGGTGGCTACAATTGGAAGGA | 207 |
|  |  | RK10-R | ATTCAATCCCACGCCCTACT |  |
| XM_010661810.1 | ethylene-responsive transcription factor 1B | ETF-F | TGGCCGACGACTCCTATATG | 162 |
|  |  | ETF-R | GTTCCCAACCAAACCCTCAC |  |
